# Multiwavelength Optical Pulse-Induced Temporal State Mapping in an ITO/SnO_2_/ITO Optoelectronic Synaptic Device

**DOI:** 10.3390/nano16191268

**Published:** 2026-10-08

**Authors:** Senyu Zhuang, Zhuang Hou, Shuqi Teng, Yingqi Zhao, Yongqi Song, Xuanyu Shan, Meng Qi, Mingan Luan, Ye Tao

**Affiliations:** 1 State Key Laboratory of Integrated Optoelectronics, Key Laboratory of UV Light-Emitting Materials and Technology of Ministry of Education, Northeast Normal University, Changchun 130024, China; 2 Laboratory of Neuromorphic Memristor Computing, College of Physical Science and Technology, Bohai University, Jinzhou 121000, China

**Keywords:** SnO_2_, optoelectronic synaptic device, paired-pulse facilitation, short-term history dependence, device-state mapping, temporal state encoding

## Abstract

A vertical ITO/SnO_2_/ITO optoelectronic synaptic device was fabricated by magnetron sputtering for optical sensing and temporal-state encoding. The ITO/SnO_2_ stack exhibited 88.3% average transmittance over 400–800 nm. The device responded to 380, 550 and 660 nm illumination and showed paired-pulse facilitation (PPF) together with pulse-width, interval and number-dependent modulation, evidencing finite-timescale history dependence and pulse-dependent accumulation. These dynamics are consistent with rapid photocarrier excitation followed by slower localized-state trapping, detrapping, redistribution and relaxation, detrapping, redistribution and relaxation. All 16 possible 4-bit optical sequences were experimentally applied and identical pulse numbers arranged at different temporal positions produced different final current states. The measured state library was used for visual feature construction: a simple multilayer perceptron achieved 96.06 ± 0.19% test accuracy for MNIST over five independent runs, while a hybrid representation achieved 98.26 ± 0.28% over five independent runs for four-frame trajectory recognition; ablation showed that device-state features preserved temporal-order information. These results support temporal-order-sensitive physical encoding in a simple transparent oxide device.

## 1. Introduction

Machine vision and edge sensing generate increasing volumes of data that must be transferred between physically separated sensors, memory and processors, adding latency and energy cost [1,2]. Near- and in-sensor computing reduce this burden by moving information processing toward the sensing front end [1]. Memristive crossbar array-based hardware has also been demonstrated for compressed sensing and event-driven neuromorphic processing, providing an efficient route for real-time sensing-signal processing at the edge [3]. Optoelectronic synaptic devices are attractive because optical stimuli can directly modulate current or conductance, combining sensing, short-term state retention and feature transformation in one platform [4].

Reservoir computing (RC) is particularly suited to temporal information because a nonlinear dynamical system with fading memory transforms time-dependent inputs into a state space that can be decoded by a comparatively simple readout [5,6]. Physical RC transfers this input-to-state transformation from a software network to the intrinsic dynamics of a material or device [6]. In optoelectronic devices, photocarrier generation, trapping, release, redistribution and relaxation occur over finite timescales. The instantaneous electrical state can therefore retain partial information from earlier optical stimuli, providing the short-term memory and nonlinearity required for temporal encoding without recurrently training the physical medium itself.

Optoelectronic devices have been used to map optical pulse sequences to physical states for image, motion and speech processing [7,8,9,10]. Many demonstrations, however, rely on heterojunctions, multilayer stacks, or multiple physical outputs. A simpler transparent oxide structure is useful for isolating how much temporal processing can arise from the carrier kinetics of a single functional layer.

SnO_2_ is a transparent wide-bandgap semiconductor whose optoelectronic properties are sensitive to defects and localized states. SnO_2_ devices have shown synaptic plasticity, optical conductance modulation and handwritten-digit recognition [11,12], while sub-bandgap persistent photoconductivity has been associated with defect-related states and slow carrier relaxation [13].

For temporal-state mapping, the essential requirement is not simply a large photoresponse or multiple static conductance levels, but an output that depends on the temporal arrangement of preceding stimuli. In particular, if sequences containing the same number of identical optical pulses produce different final states when the pulse positions differ, the device retains information about temporal order rather than merely integrating total illumination. Although synaptic plasticity and optical recognition have been reported in SnO_2_ systems [11,12], the connection among multiwavelength transient response, finite-time relaxation, temporal-order-sensitive multibit encoding and subsequent visual feature construction remains insufficiently explored in a simple vertical ITO/SnO_2_/ITO structure.

Here, a vertical ITO/SnO_2_/ITO device was characterized from multiwavelength photoresponse through PPF and pulse-dependent state modulation. All 16 possible 4-bit optical sequences were experimentally applied to establish a temporal state library, which was then reused for MNIST and four-frame trajectory recognition with simple MLP readouts. The study therefore links transient carrier dynamics to temporal-order-sensitive physical encoding and visual information processing.

## 2. Materials and Methods

### 2.1. Device Fabrication

Vertical ITO/SnO_2_/ITO devices were fabricated by magnetron sputtering on commercial ITO-coated glass. Substrates were ultrasonically cleaned in trichloroethylene, acetone, ethanol and deionized water for 10 min each and dried with N_2_.

SnO_2_ was deposited from a 99.99% target in Ar after 5 min pre-sputtering at 88 W and 7.5 mTorr for 10 min, yielding a 60–70 nm film.

The top ITO electrode was deposited from a 99.99% In_2_O_3_:SnO_2_ (90:10 by mass) target after 5 min pre-sputtering at 60 W and 3.5 mTorr for 6 min, with Ar and 50 sccm O_2_. Circular electrodes (0.1 mm diameter) were patterned through a shadow mask. No intentional heating or post-deposition annealing was used.

### 2.2. Material Characterization

Optical transmittance and absorption of the ITO/SnO_2_ stack were measured from 300–1000 nm using a UH4150 UV–Vis–NIR spectrophotometer. An apparent stack-level optical transition energy was estimated from a direct-transition Tauc plot of (αhν)^2^ versus hν.

SnO_2_ morphology was measured by AFM over 30 μm × 30 μm. XPS, calibrated to C 1s, was used to analyze Sn 3d and O 1s states; film thickness was measured by profilometry.

### 2.3. Electrical and Optoelectronic Measurements

Electrical and optoelectronic measurements were performed in air at room temperature using a Keithley 2636B and a probe station, with the top ITO connected to the positive terminal. Current–voltage characteristics were recorded for 10 consecutive 0 → +2 → 0 V and 0 → −2 → 0 V cycles.

Dynamic optical measurements used a constant 100 mV read voltage. Optical stimuli were generated by a xenon lamp (LA-410UV, Hayashi) with wavelength-selective filters and were incident on the device surface. Responses to 380, 550 and 660 nm were compared at an incident power density of 78.3 mW cm^−2^. The corresponding photon flux was calculated as Φ = Pλ/hc. Additional measurements varied power density and pulse timing to examine intensity dependence, PPF, pulse-width effects, inter-pulse relaxation, and pulse-number-dependent accumulation. The experiment-specific pulse conditions are reported with the corresponding figures.

### 2.4. Device-State Mapping and Pattern Recognition

For 4-bit temporal encoding, “0” denotes no illumination and “1” denotes 380 nm illumination. All 16 sequences from [0000] to [1111] were experimentally applied. Repeated 16-state measurements were performed using independently randomized sequence orders. Repeatability of the 16-state mapping was evaluated by three repeated measurements on one device and measurements across three independently fabricated devices. The pre-sequence current was used as the baseline and the current change at the end of the fourth-bit window defined the corresponding device state, establishing a measured lookup relation between each 4-bit code and one scalar current-state value.

For MNIST, binarized 28 × 28 images were divided into 2 × 2 blocks; each 4-bit block code was mapped to a measured state, yielding a standardized 196-dimensional vector for MLP classification. For comparison, raw 784-dimensional binary images, 196-dimensional 2 × 2 average-pooled images, conventional 2 × 2 binary encoding, and non-device-derived software lookup representations were evaluated. The same training protocol was used for all representations, and five independent runs were performed for each condition.

For trajectory recognition, four consecutive 28 × 28 binary frames were encoded pixel-wise as 4-bit temporal codes and mapped to a 28 × 28 device-state map. The flattened map was combined with auxiliary spatiotemporal features and classified by an MLP into left, right, up, down and stay. Additional ablation studies were performed for device-state, auxiliary, and combined features, together with conventional software temporal encodings; five independent runs were conducted for each condition.

## 3. Results and Discussion

### 3.1. Device Architecture and Material Characterization

**Figure 1:**
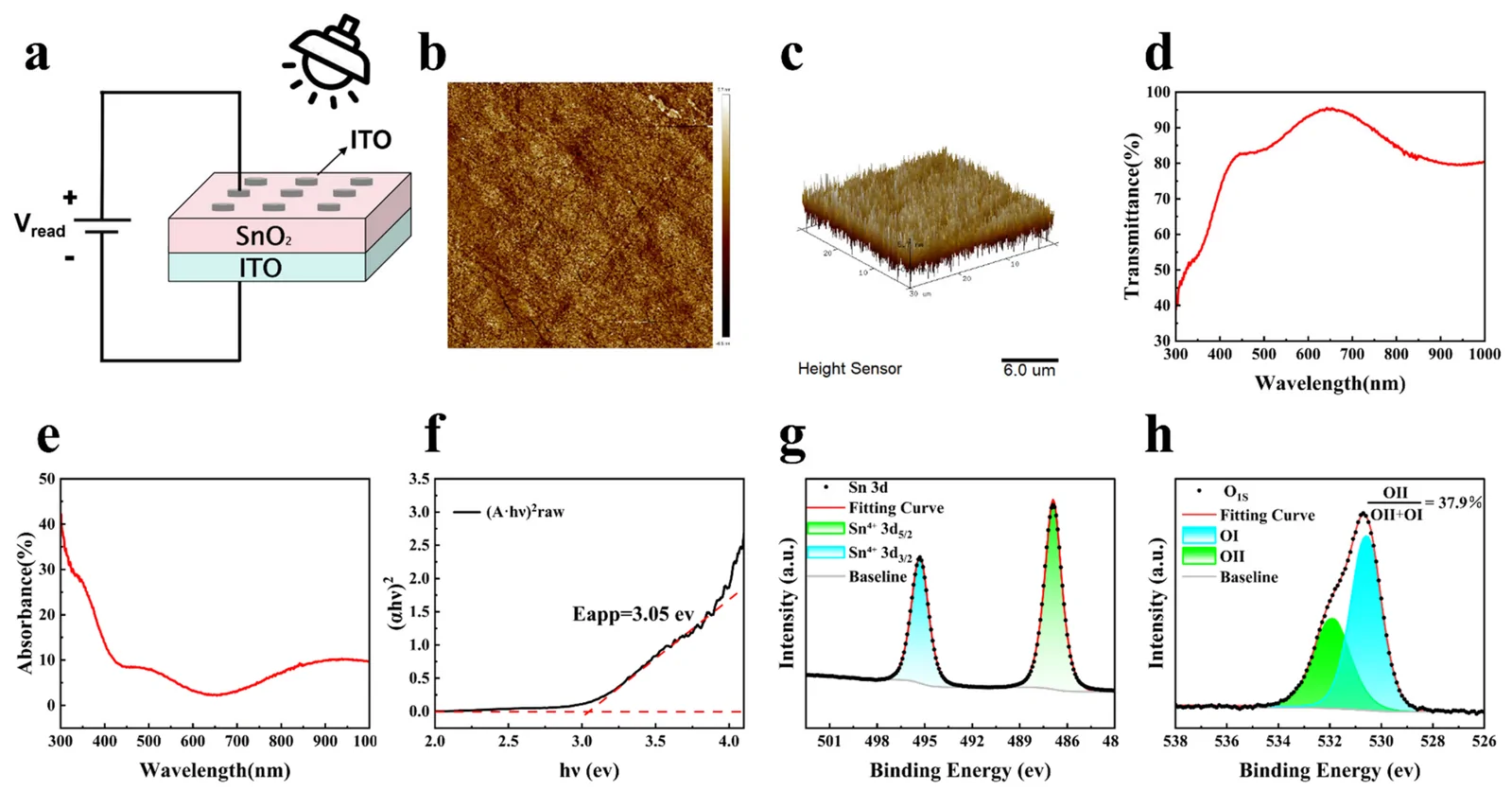
Device architecture and material characterization. (**a**) ITO/SnO_2_/ITO structure. (**b**,**c**) AFM images. (**d**,**e**) Transmittance and absorption. (**f**) Tauc plot. (**g**,**h**) Sn 3d and O 1s XPS spectra.

Figure 1a shows the vertical ITO/SnO_2_/ITO structure, with a 60–70 nm SnO_2_ functional layer between transparent ITO electrodes, enabling top-side optical excitation and vertical electrical measurement.

AFM images (Figure 1b,c) show a continuous fine-grained SnO_2_ surface without obvious large defects. Over 30 μm × 30 μm, Rq and Ra were 2.62 and 1.31 nm, respectively [14].

The ITO/SnO_2_ stack showed 88.3% average transmittance over 400–800 nm (Figure 1d), with 64.1%, 88.3%, and 95.0% at 380, 550 and 660 nm, respectively. Absorption decreased toward the visible region (Figure 1e), confirming high transparency while retaining wavelength-dependent optical interaction [14,15].

A direct-transition Tauc analysis of the ITO/SnO_2_ stack yielded an apparent transition energy of 3.05 eV with R^2^ ≈ 0.9984 (Figure 1f). Because the optical contribution of the ITO substrate was not subtracted, this value should not be interpreted as the intrinsic bandgap of the SnO_2_ film and is retained only as a descriptive optical parameter of the measured stack [14,16,17,18].

XPS further characterized the chemical environment of the SnO_2_ film (Figure 1g,h). The Sn 3d_5/2_ and Sn 3d_3/2_ peaks at 486.8 and 495.2 eV are consistent with predominantly Sn^4+^ [19]. The O 1s spectrum was fitted with a lattice-oxygen component at 530.5–530.7 eV and higher-binding-energy non-lattice component at 531.7–532.0 eV. The latter accounted for 37.9% of the fitted O 1s area and may include defect-related as well as surface-related oxygen environments; it is therefore not treated as a direct quantitative measure of oxygen-vacancy concentration [17,18,19,20].

Together, the optical and XPS results support consideration of localized defect-related states in the dynamic photoresponse.

### 3.2. Electrical Characteristics and Multiwavelength Photoresponse

**Figure 2:**
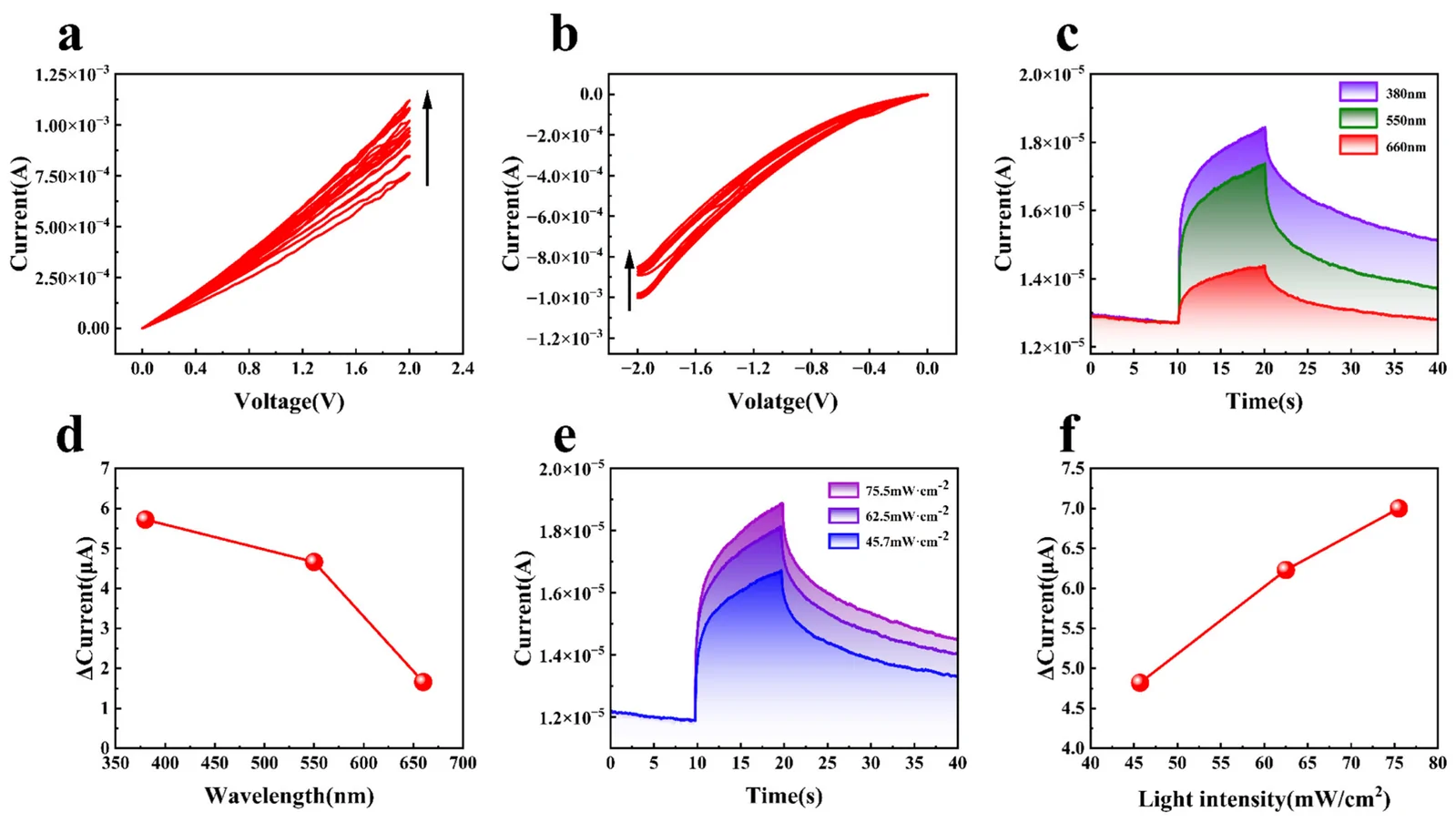
Electrical and photoresponse characteristics. (**a**,**b**) Repeated positive and negative voltage sweeps. (**c**,**d**) Representative dynamic responses and extracted Δ*I* values at 380, 550 and 660 nm. (**e**,**f**) 380 nm responses and Δ*I* versus incident power density.

Figure 2a,b show bias-history-dependent modulation. During repeated positive 0 → +2 → 0 V sweeps, current generally increased with cycle number, whereas the absolute current decreased over repeated negative sweeps, indicating conductance dependence on prior electrical stimulation. Although both electrodes are ITO, the bottom ITO/SnO_2_ and SnO_2_/top-ITO interfaces are formed by different deposition sequences and may therefore be electronically nonequivalent. The electrical hysteresis is therefore described phenomenologically as bias-history-dependent trapping/detrapping with possible field-induced ionic, defect, or interfacial redistribution.

The optical dynamics were then examined at a 100 mV read voltage. At this low fixed bias, the current is treated as a conductance-state readout without assigning a unique transport mechanism. Under 10 s illumination at 380, 550 and 660 nm with the same incident power density of 78.3 mW cm^−2^, the current rose rapidly at light onset and then continued to evolve more slowly during illumination (Figure 2c). After the light was removed, the current dropped rapidly but recovered only gradually toward its initial dark level. This two-stage recovery indicates that the optically induced state does not disappear instantaneously and instead persists over a finite relaxation timescale [21,22].

Using the pre-illumination dark current I_dark_ and peak illumination current I_peak_, the photoinduced change was defined as

Δ*I = I_peak_ − I_dark_*.

The resulting Δ*I* values were approximately 5.7, 4.7 and 1.7 μA for 380, 550 and 660 nm, respectively (Figure 2d), giving the order 380 > 550 > 660 nm. Independent repeated measurements yielded 5.73 ± 0.04, 4.67 ± 0.04 and 1.69 ± 0.03 μA, respectively (mean ± SD, *n* = 3), confirming the same wavelength-dependent trend. After photon-flux normalization, the responses were 3.81, 2.17 and 0.65 μA per 10^17^ photons cm^−2^ s^−1^ at 380, 550 and 660 nm, respectively, retaining the 380 > 550 > 660 nm trend (Table S2). In contrast, a bottom-ITO/substrate control showed non-systematic relative current variations under 380, 550 and 660 nm illumination, without a reproducible wavelength-dependent photoresponse (Table S5).

The clear responses at 550 and 660 nm demonstrate visible-light sensitivity, while their microscopic origin cannot be determined from the stack-level Tauc analysis alone.

Under 380 nm illumination, increasing the incident power density from 45.7 to 62.5 and 75.5 mW cm^−2^ increased the response while preserving the rapid-rise/slow-evolution profile and post-illumination relaxation (Figure 2e) [13,16,17,18].

In the representative dataset shown in Figure 2f, Δ*I* increased from 4.8 to 6.2 and 7.0 μA as the incident power density increased from 45.7 to 62.5 and 75.5 mW cm^−2^. Across three repeated measurements, the corresponding Δ*I* values were 4.85 ± 0.09, 6.20 ± 0.08, and 7.00 ± 0.08 μA, respectively (mean ± SD, *n* = 3), confirming the same intensity-dependent trend [21,22,23].

These multiwavelength and intensity-dependent dynamics provide the basis for the history-dependent behavior examined next.

### 3.3. Short-Term History-Dependent Dynamics

**Figure 3:**
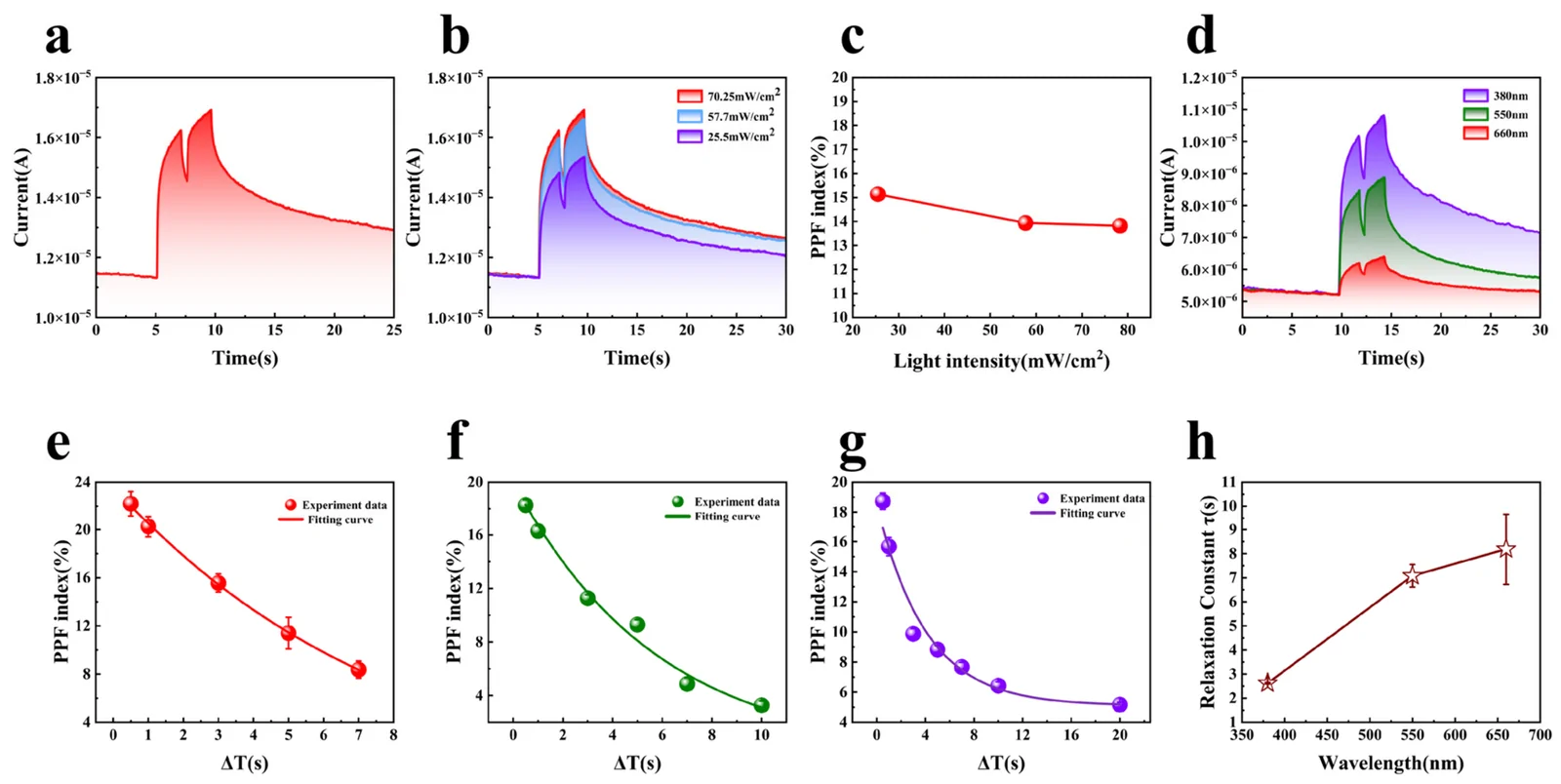
Paired-pulse facilitation. (**a**) Representative 380 nm response. (**b**,**c**) Power-density dependence. (**d**) Wavelength dependence. (**e**–**g**) PPF index versus interval shown as mean ± SD (*n* = 3), with exponential fits to the mean values. (**h**) Relaxation time τ obtained from independent fitting of three repeated datasets, shown as mean ± SD (*n* = 3).

PPF was first examined using two identical 380 nm pulses at 78.3 mW cm^−2^, with a pulse width of 2 s, an inter-pulse interval Δt = 0.5 s and a 100 mV read voltage (Figure 3a). After the first pulse, the current decayed but did not fully return to the initial dark level before the second pulse arrived. The second stimulus therefore acted on a residual nonequilibrium state and produced a larger peak response, directly demonstrating short-term history dependence [24,25,26].

Using the initial dark current I_0_, the first- and second-pulse amplitudes were

*A*_1_ = *I*_1_ − I_0_,

*A*_2_ = *I*_2_ − I_0_.

and the PPF index was calculated as

$$\mathrm{PPF}\;\mathrm{index}=\frac{{A_{2}-A}_{1}}{A_{1}}\times 100\%$$

With A_1_ and A_2_ referenced to the same initial dark current, A_2_ > A_1_ quantifies the enhancement of the second response caused by the residual state.

In the representative dataset shown in Figure 3b,c, the PPF index changed only slightly from 15.1% to 13.9% and 13.8% as the incident power density increased from 25.5 to 57.7 and 70.25 mW cm^−2^. Across three repeated measurements, the corresponding PPF indices were 15.27 ± 0.38%, 13.95 ± 0.07% and 13.86 ± 0.10%, respectively (mean ± SD, *n* = 3), confirming the same weak power-density dependence. Response magnitude and relative facilitation therefore quantify different aspects of the dynamics [27].

PPF was also observed at 550 and 660 nm (Figure 3d), showing that the finite-timescale history dependence extends across the tested wavelengths [13,16,21]. The PPF index decreased monotonically with Δt (Figure 3e–g). At 660 nm, the PPF index decreased from 22.20 ± 1.02% at 0.5 s to 8.37 ± 0.71% at 7 s. At 550 nm, it decreased from 18.27 ± 0.40% at 0.5 s to 3.27 ± 0.31% at 10 s. At 380 nm, it decreased from 18.73 ± 0.55% at 0.5 s to 5.17 ± 0.15% at 20 s (mean ± SD, *n* = 3). Longer intervals allow more relaxation before the second stimulus [24,26,28].

The mean interval-dependent PPF values in Figure 3e–g were fitted using

$$\mathrm{PPF}(\Delta t)=P_{\infty }+A\;\exp(-\frac{\Delta t}{\tau }),$$

Here, P∞ is the long-interval residual component, A the decay amplitude, and τ the characteristic relaxation time. To evaluate the reproducibility of τ, each of the three repeated datasets was fitted independently. The resulting relaxation times were 8.19 ± 1.47 s at 660 nm, 7.08 ± 0.47 s at 550 nm and 2.62 ± 0.03 s at 380 nm (mean ± SD, *n* = 3).

Accordingly,

τ_660_ > τ_550_ > τ_380_.

Although 380 nm produced the largest absolute response, it had the shortest fitted τ. Response amplitude and memory timescale are therefore not directly correlated. The wavelength dependence of τ may reflect different relative contributions of photocarrier generation, localized-state trapping/detrapping, and subsequent recovery. The present PPF data, however, do not support assignment of these timescales to specific microscopic defect levels [24,25,26,29].

The interval dependence establishes finite-time memory; pulse-parameter measurements were then used to test how this memory controls state accumulation [30].

**Figure 4:**
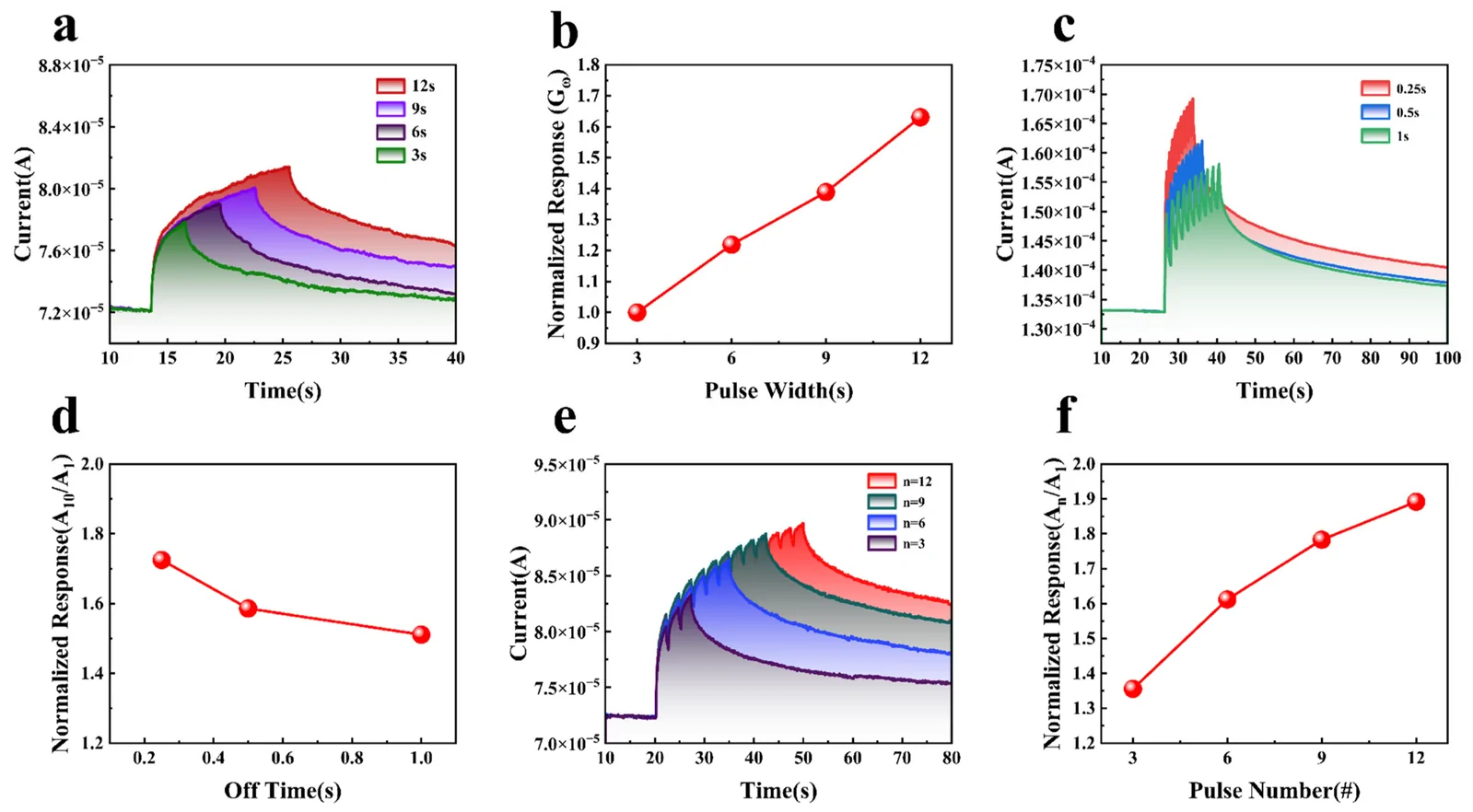
Pulse-parameter-dependent state modulation at 380 nm. (**a**,**b**) Pulse-width dependence. (**c**,**d**) Interval dependence. (**e**,**f**) Pulse-number dependence. Read voltage: 100 mV; incident power density: 78.3 mW cm^−2^ unless otherwise specified. Representative datasets are shown; quantitative results from three repeated measurements are reported in the text as mean ± SD.

Pulse duration, inter-pulse dark interval, and pulse number were varied under 380 nm illumination to probe state accumulation beyond the two-pulse PPF response (Figure 4).

For single pulses of 3, 6, 9 and 12 s, the final current change increased with duration (Figure 4a). With Δ*I_w_ = I_end,w_* − *I_0,w_* and normalization to the 3 s response,

Δ*I_w_ = I_end,w_ − I*_0,*w*_.

$$R_{w}=\frac{\Delta I_{w}}{\Delta I_{3s}}.$$

Across three repeated measurements, the normalized responses at 6, 9 and 12 s were 1.213 ± 0.006, 1.385 ± 0.006 and 1.627 ± 0.004, respectively (mean ± SD, *n* = 3), with the 3 s response defined as 1.00 (Figure 4b). The monotonic increase shows that pulse duration alone can tune the final device state. A longer pulse provides more time for photocarrier generation and slower localized-state evolution during illumination, producing a larger current change [27,31,32,33].

For ten consecutive 0.5 s pulses, reducing the dark interval toff from 1 to 0.5 and 0.25 s strengthened current accumulation (Figure 4c), consistent with less recovery between stimuli.

Using the first- and tenth-pulse amplitudes relative to I_0_,

A_1_ = I_1_ − I_0_,

A_10_ = I_10_ − I_0_.

In the representative dataset shown in Figure 4d, the accumulation ratio A_10_/A_1_ increased from 1.51 to 1.59 and 1.72 as t_off_ decreased from 1 to 0.5 and 0.25 s. Across three repeated measurements, the corresponding ratios were 1.510 ± 0.011, 1.584 ± 0.005 and 1.724 ± 0.012, respectively (mean ± SD, *n* = 3), confirming the same interval-dependent trend (Figure 4d). This trend directly links the multi-pulse response to the relaxation behavior observed in PPF: reducing the available recovery time allows residual carrier/state occupation from earlier stimuli to contribute more strongly to subsequent responses [24,26,28].

Pulse-number dependence was tested with trains of 3, 6, 9 and 12 pulses (2 s width, 0.5 s interval). The final current increased progressively with pulse number (Figure 4e).

With A_1_ and A_n_ defined relative to the same initial current,

A_1_ = I_1_ − I_0_,

*A_n_ = I_n_ −* I_0_,

In the representative dataset shown in Figure 4f, the normalized accumulation A_n_/A_1_ reached 1.36, 1.61, 1.78, and 1.89 for 3, 6, 9 and 12 pulses, respectively. Across three repeated measurements, the corresponding values were 1.346 ± 0.012, 1.620 ± 0.037, 1.783 ± 0.008, and 1.896 ± 0.013 (mean ± SD, *n* = 3), confirming the same pulse-number-dependent trend.

The incremental increase became progressively smaller: the change in normalized response was 0.25 from 3 to 6 pulses, 0.17 from 6 to 9 and 0.11 from 9 to 12. This behavior indicates nonlinear accumulation with a tendency toward a dynamic balance between continued excitation and simultaneous relaxation [32]. It does not, however, demonstrate that a fully saturated state was reached within 12 pulses. Operational stability was further evaluated using 10 repeated 10-pulse trains. The normalized final response remained within 92.47–103.61% of the initial value, with a coefficient of variation of 3.59% (Figure S3a). Repeated [1111] measurements gave an average recovery-to-baseline time of 78.8 ± 5.6 s (mean ± SD, *n* = 10); indicating a reproducible finite recovery timescale.

Thus, the device output depends on both the present stimulus and residual states from earlier inputs, providing the finite-time nonlinear memory required for multibit temporal mapping.

### 3.4. Mechanism of Dynamic Photoresponse and History Dependence

**Figure 5:**
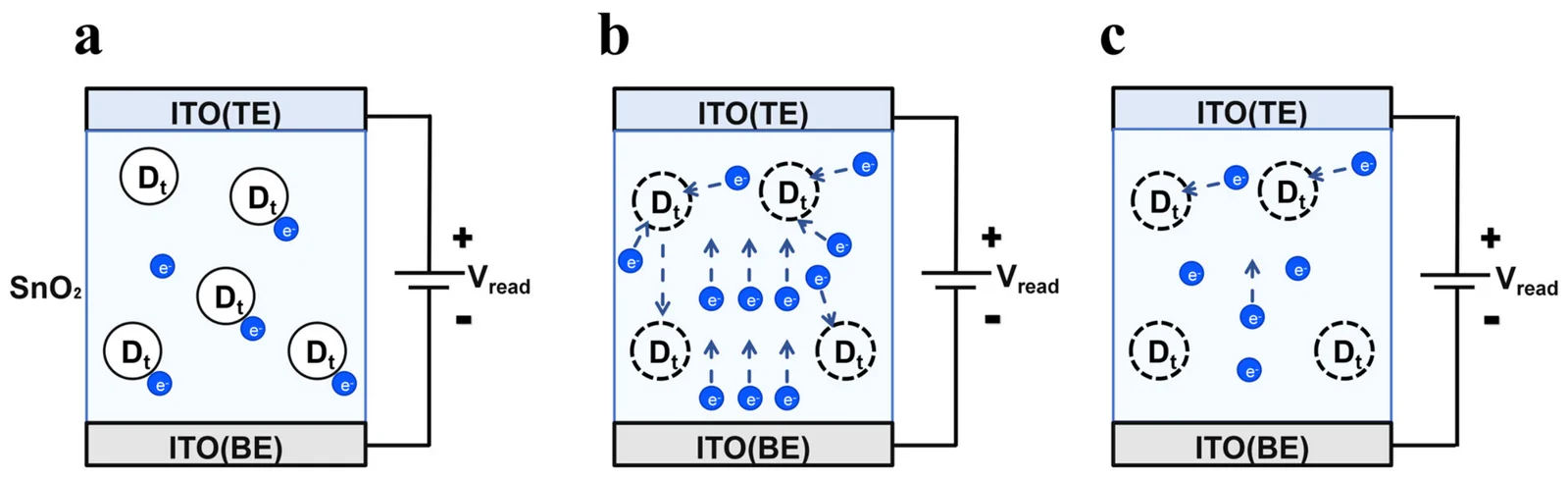
Proposed carrier-dynamic framework. (**a**) Dark state. (**b**) Rapid photoexcitation plus slower localized-state redistribution under illumination. (**c**) Post-illumination relaxation leaving a finite-lived residual state. Dt denotes a generic localized-state site. The repeated Dt symbols schematically represent multiple localized sites within the SnO_2_-containing active region and do not indicate different defect species or distinct energy levels.

Figure 5 summarizes a carrier-dynamic framework consistent with the optical, XPS, photoresponse, and PPF results; it is not intended as a unique identification of specific defect species or energy levels.

The high-binding-energy O 1s component and the slow photoresponse/recovery are consistent with localized-state-assisted dynamics in the SnO_2_-containing active region, potentially including interface-related states [13,16,18,20,21,34]. Because the measurements were performed in ambient air, surface oxygen adsorption/desorption may also participate in the photoresponse. Adsorbed oxygen species on SnO_2_ can capture electrons and modify the near-surface carrier concentration, while illumination-induced changes in oxygen adsorption or charge state may contribute to the slow evolution and recovery of the conductance [18]. Because the O 1s component may include both defect-related and surface-related environments, its fitted fraction is not treated as a direct oxygen-vacancy concentration.

In darkness (Figure 5a), multiple localized-state sites, schematically denoted by Dt, may be occupied or unoccupied and influence subsequent excitation, trapping, release and recovery [34].

Upon illumination (Figure 5b), the rapid initial current rise is attributed primarily to fast photocarrier generation. For 380 nm excitation, the photon energy is 3.26 eV. Because the 3.05 eV Tauc value was obtained from the complete ITO/SnO_2_ stack, it is not used here to assign the 380 nm response to an intrinsic SnO_2_ band-edge transition.

The slower increase during sustained illumination can arise from processes that evolve on longer timescales than initial photogeneration [35,36,37], including defect-assisted excitation, detrapping, and redistribution of carriers among localized states. The experimentally observed fast-rise/slow-accumulation profile is therefore consistent with a superposition of a rapid excitation process and slower localized-state evolution rather than a single instantaneous mechanism.

Clear photoresponses were also observed at 550 and 660 nm. Together with the slow recovery and the presence of non-lattice oxygen environments, these responses are consistent with contributions from localized-state-assisted processes; however, the present data do not determine the energetic positions or microscopic identities of these states.

After the optical stimulus is removed (Figure 5c), photogeneration ceases and the current first decreases rapidly before recovering more slowly. The fast component can be associated with rapid loss of mobile photoexcited carriers, whereas the slower component may involve recombination, retrapping, redistribution of localized-state occupation, and recovery of surface oxygen adsorption/charge equilibrium in ambient air [18,35,36,37,38]. Because controlled-atmosphere measurements were not performed, the relative contributions of intrinsic localized-state dynamics and surface oxygen-mediated processes cannot be separated in the present study. The resulting residual nonequilibrium state persists for a finite time and provides a physical basis for short-term memory.

A second pulse arriving before complete recovery therefore produces A_2_ > A_1_, whereas increasing Δt allows more relaxation and reduces the PPF index [39].

The same kinetic competition explains the broader pulse-parameter trends: longer pulses permit further state evolution during illumination, shorter dark intervals leave less time for recovery and additional pulses accumulate on progressively modified initial states. The decreasing increment at high pulse numbers reflects the simultaneous action of accumulation and relaxation.

Overall, the finite memory is consistent with optically driven carrier/localized-state dynamics in the SnO_2_-containing active region. However, because the measurements were performed in ambient air, surface oxygen adsorption/desorption may also contribute to the slow conductance evolution and recovery. Possible interfacial, bias-induced, and minor photothermal contributions likewise cannot be completely excluded.

### 3.5. 4-Bit Optical Input Encoding and Device-State Mapping

The finite relaxation and pulse-dependent accumulation above allow the device to act as a temporal encoder whose current depends on both the present input and residual states from preceding stimuli. Complete 4-bit binary optical sequences were therefore applied to test physical input-to-state transformation [7,40,41,42,43,44,45].

Binary “0” and “1” denote no illumination and 380 nm illumination at 78.3 mW cm^−2^, respectively. A 100 mV read voltage was applied throughout the experiment; each bit lasted 2 s and adjacent bits were separated by 0.5 s. A 4-bit input has 2^4^ = 16 possible sequences and every sequence from [0000] to [1111] was physically applied to the device rather than generated by interpolation, fitting or numerical simulation. Before each sequence, the device was allowed to recover close to its pre-sequence baseline rather than using a fixed waiting interval. Independent [1111] measurements gave an average recovery-to-baseline time of 78.8 ± 5.6 s (Figure S3b).

**Figure 6:**
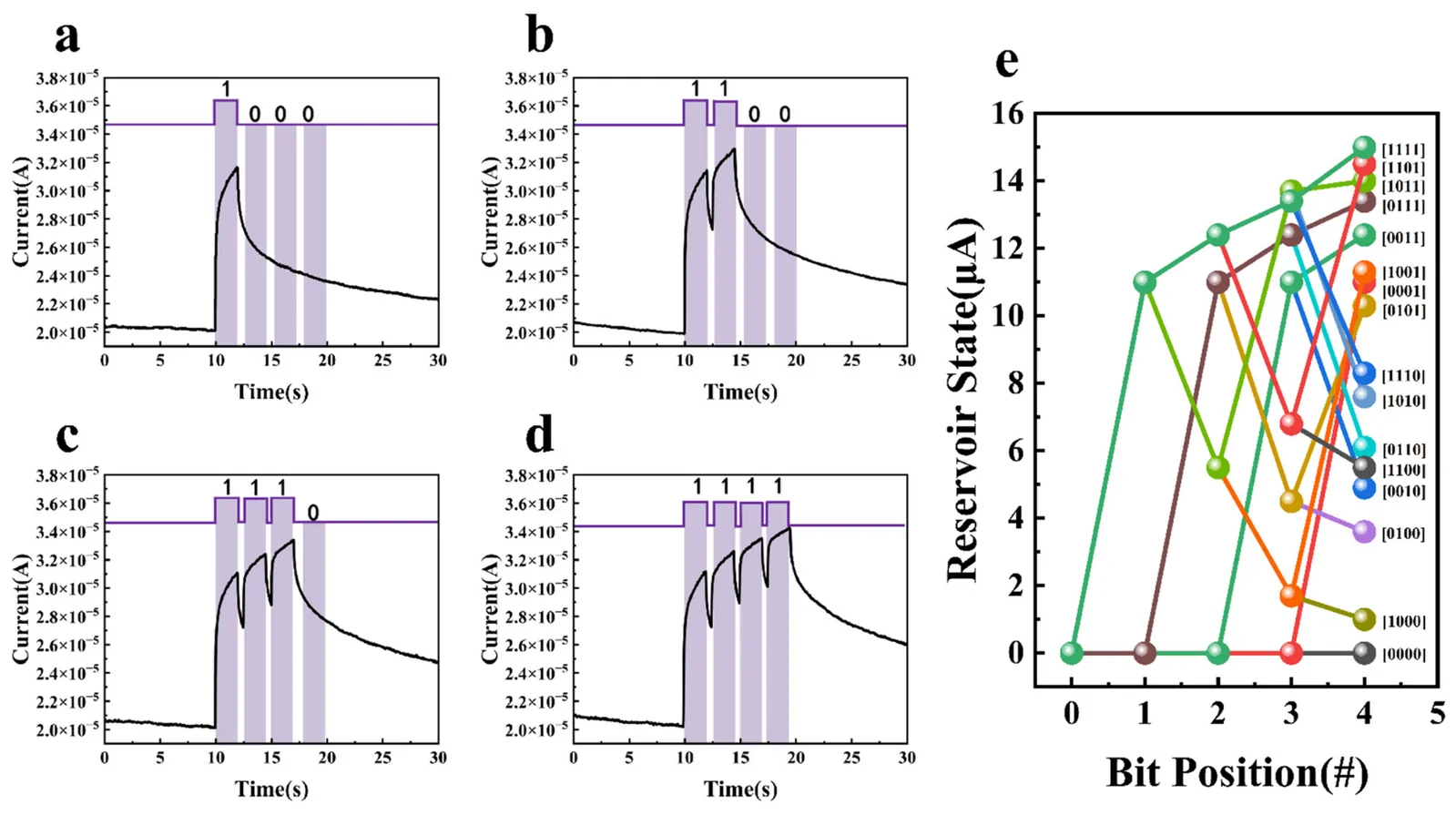
Four-bit optical encoding and device-state mapping. (**a**–**d**) Responses to [1000], [1100], [1110], and [1111]. (**e**) Measured evolution for all 16 sequences; fourth-bit values define the state library.

Figure 6a–d show [1000], [1100], [1110] and [1111]. Because the current does not fully recover between closely spaced pulses, each subsequent “1” interacts with the residual state left by earlier inputs, producing stronger accumulation for consecutive pulses.

For sequence s, the device state R_s_ was defined as

*R_s_ = I_final,s_* − I_0,s_,

The sequence-specific baseline reduces slow baseline variation while keeping the sampling point identical across all four-bit inputs. Baseline tracking showed no systematic monotonic drift within the randomized measurement cycles (Figure S2).

The [0000] sequence was measured as the zero reference; this relative reference does not imply perfectly drift-free dark current.

Figure 6e summarizes the measured current-state evolution for all 16 sequences. Different temporal inputs yield different final current changes, demonstrating that a single physical device can transform a finite set of binary histories into sequence-dependent states. Repeatability measurements showed consistent sequence-dependent state distributions within one device and across three devices (Figure S1a,b). Among the 15 neighbouring state pairs ordered by mean response, 11 showed non-overlapping mean ± SD intervals, while four showed partial overlap.

Temporal-order sensitivity is clearest for [1000], [0100], [0010], and [0001], which contain one identical pulse at different positions. An early pulse has more time to relax before final sampling, whereas a late pulse leaves a larger residual response. Similar position-dependent differences occur for sequences containing two or three “1” bits. Thus, the final state depends on temporal arrangement and recent history, not only on total pulse number or illumination duration.

This behavior follows directly from the PPF relaxation and nonlinear accumulation in Section 3.3: each input changes the state on which the next input acts.

The device therefore performs a physical temporal input-to-state transformation rather than simple unweighted summation [40,41,42].

The 16 experimentally measured states therefore form a compact physical encoding library linking 4-bit optical histories to current-state values. In the following visual tasks, this measured input-to-state relation is reused by software lookup to process many samples, while the physical device supplies an experimentally measured, history-dependent state library and the final classification remains computational [40,41,42,43,44,45]. This distinction avoids implying fully online hardware processing of the complete datasets.

### 3.6. Visual Information Processing

#### 3.6.1. Handwritten-Digit Recognition

For MNIST, each 28 × 28 image was binarized at a grayscale threshold of 80 and divided into non-overlapping 2 × 2 blocks. The four pixels in each block formed a 4-bit code, giving 196 local units per image [46].

Each code was replaced by its experimentally measured state, converting the image into a 196-dimensional device-state vector.

For block k with code s_k_,

R_k_ = R(s_k_),

and the image representation was

R = [R_1_,R_2_,…,R_196_].

Thus, local spatial patterns were represented by experimentally grounded feature values [7,41,43,44,47].

A representative measured 16-state library was reused by software lookup across MNIST rather than physically applying all 70,000 images.

Each feature dimension was standardized using training-set statistics, which were also applied to the test set.

$$X^{\prime }i,j=\frac{X_{i,j}-\mu_{j}}{\sigma_{j}+10^{-6}},$$

The same training-set statistics were applied to the test set.

The standardized vectors were classified by a 196 → 50 → 10 MLP with ReLU hidden units and softmax output. Training used SGD (learning rate 0.002, batch size 128) and multiclass cross-entropy on the standard 60,000/10,000 MNIST split. The simple readout keeps the evaluation focused on the device-state representation rather than classifier complexity.

**Figure 7:**
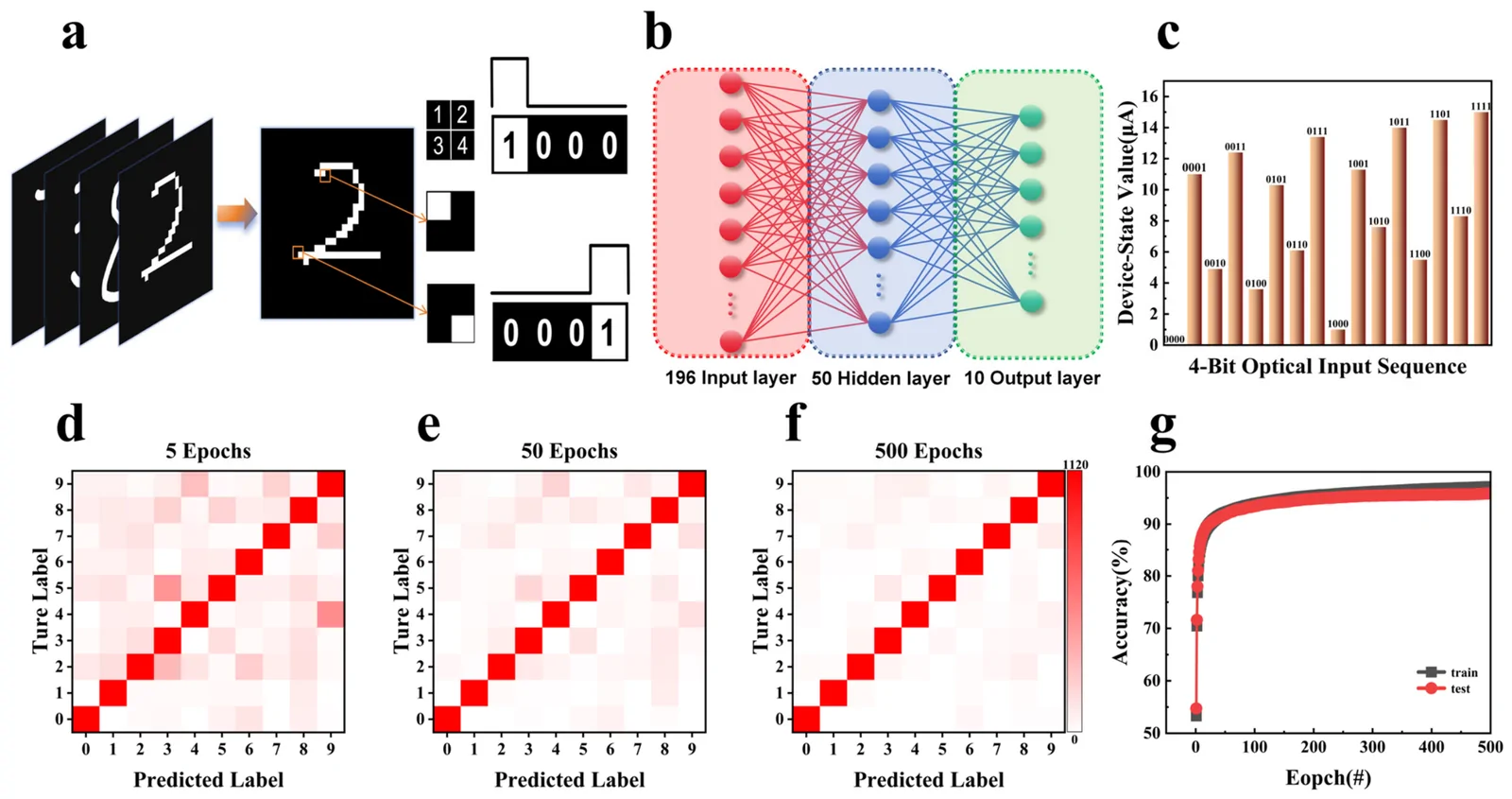
MNIST recognition. (**a**) Binarization, 2 × 2 encoding, and state lookup. (**b**) 196 → 50 → 10 MLP. (**c**) Measured state library. (**d**–**f**) Representative test confusion matrices. (**g**) Representative accuracy versus epoch.

Test accuracy increased from 83.16% at 5 epochs to 91.83% at 50 epochs and 95.89% at 500 epochs; final training accuracy was 97.19% (Figure 7d–g). Across five independent runs, the measured device-state representation achieved 96.062 ± 0.188% test accuracy, comparable to the 196-dimensional software baselines (Table S3). Thus, the MNIST results demonstrate a competitive experimentally measured feature representation rather than a clear accuracy advantage from device nonlinearity.

The MNIST accuracy therefore characterizes the combined device-state mapping and software readout rather than fully online hardware recognition.

#### 3.6.2. Temporal Trajectory Recognition

The same library was then applied to a task that directly uses temporal history: the four bits represent one pixel across four successive frames rather than four neighboring spatial pixels [48,49,50,51].

Five classes were considered: left, right, up, down and stay. Each sample contained four 28 × 28 binary frames (T_1_–T_4_). At each position (x,y), the time-ordered pixel values formed

S(x,y) = [b_1_,b_2_,b_3_,b_4_],

where b_1_–b_4_ are the pixel states at T_1_–T_4_.

Each temporal code was mapped to its measured device state:

R(x,y) = R[S(x,y)].

Mapping all 28 × 28 temporal codes generated a device-state map in which each pixel represents its local four-frame history [48,49,50,51].

As for MNIST, the measured state library was reused by software lookup across the synthetic dataset.

Flattening the state map produced 784 device-derived temporal features. To construct the final classifier input, 64 spatial-projection/statistical features were extracted from the state map and 20 centroid-motion features from the original sequence, yielding an 868-dimensional hybrid vector. The device-derived component therefore accounts for 784 of the 868 input dimensions.

An 868 → 80 → 5 MLP with ReLU and softmax served as the readout.

The controlled synthetic dataset contained 4000 training and 1000 test samples with balanced representation of the five classes. The task is therefore intended as a proof-of-concept test of temporal feature construction under controlled conditions rather than as a demonstration of online recognition in complex real-world video scenes.

**Figure 8:**
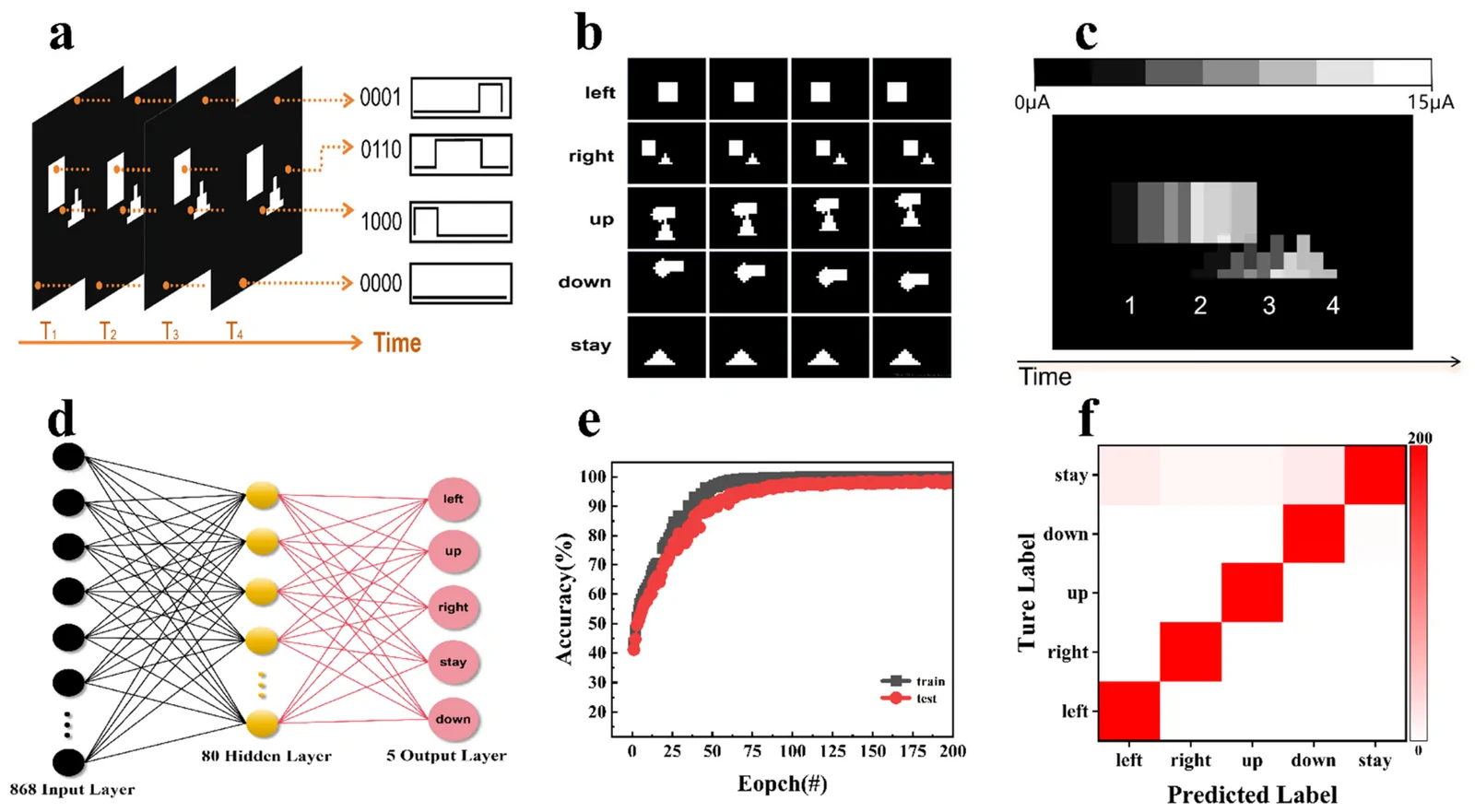
Temporal trajectory recognition. (**a**) Pixel-wise 4-bit temporal encoding. (**b**) Five representative motion classes. (**c**) Device-state map. (**d**) 868 → 80 → 5 MLP. (**e**) Accuracy. (**f**) Test confusion matrix.

In the representative run shown in Figure 8, test accuracy increased from 52.8% at 5 epochs to 90.5% at 50 epochs and 98.0% at 200 epochs, with 100.0% training accuracy (Figure 8e,f). This task directly uses temporal-order sensitivity. At a fixed pixel, sequences containing the same number of “1” states can map to different measured values when their temporal positions differ, because earlier optical inputs have more time to relax before the final state is sampled. Pixel-wise 4-bit mapping therefore converts distinct local motion histories into sequence-dependent device-state features.

The 98.0% test accuracy characterizes the complete hybrid framework because the classifier receives 784 device-state-derived features plus 84 auxiliary features. Ablation analysis showed that the device-state features alone achieved 85.10 ± 1.38% accuracy, compared with 99.90 ± 0.00% for the auxiliary features and 98.26 ± 0.28% for the combined representation. A conventional software temporal encoding achieved 85.42 ± 0.54%, whereas an order-insensitive pulse-count encoding achieved 58.20 ± 0.40%. These results indicate that the device-state mapping preserves temporal-order information, while the high accuracy of the hybrid model is strongly influenced by the auxiliary motion features (Table S4).

Nevertheless, compared with the spatial MNIST mapping, the trajectory task provides the more direct functional use of the device’s finite-timescale memory: temporal order at each pixel is explicitly transformed into a state value before software classification, linking the experimentally measured carrier dynamics to dynamic visual information processing.

## 4. Conclusions

A transparent vertical ITO/SnO_2_/ITO optoelectronic synaptic device exhibited multiwavelength photoresponse, PPF, and pulse-dependent modulation, revealing finite-timescale history dependence and pulse-dependent state accumulation. The dynamics are consistently described by rapid photocarrier excitation coupled to slower localized-state trapping, detrapping, redistribution and relaxation, although specific defect levels were not uniquely identified.

All 16 possible 4-bit optical sequences were experimentally applied to establish a measured state library. Sequences with identical pulse numbers but different temporal positions produced different final current states, demonstrating temporal-order-sensitive physical encoding rather than simple optical-dose integration. Reusing the measured library yielded 96.06 ± 0.19% MNIST accuracy and 98.0% hybrid trajectory accuracy, while ablation showed comparable performance between device-state and software temporal encoding.

These results demonstrate experimentally grounded temporal encoding in a simple transparent oxide device. Although within-device and device-to-device repeatability was evaluated, future work should examine long-term stability and larger device populations and pursue more direct hardware integration.

## Data Availability

The original contributions presented in this study are included in the article/Supplementary Material. Further inquiries can be directed to the corresponding authors.

## Supplementary Materials

The following supporting information can be downloaded at: https://www.mdpi.com/article/10.3390/nano16191268/s1. Figure S1a: Within-device repeatability of the 16 four-bit states over three repeated measurements. Individual symbols represent repeated measurements, and error bars denote mean ± SD; Figure S1b: Device-to-device reproducibility of the 16 four-bit states across three independently fabricated devices; Table S1: Randomized sequence-application orders used in three repeated 16-state measurement cycles; Figure S2: Baseline-current evolution during three randomized 16-state measurement cycles; Table S2: Wavelength-dependent responsivity and photon-flux-normalized response; Figure S3a: Normalized final response over 10 repeated optical pulse trains (100 optical pulses in total); Figure S3b: Recovery times obtained from 10 repeated [1111] stimulation measurements. The average recovery time was 78.8 ± 5.6 s (mean ± SD); Table S3: MNIST baseline comparison; Table S4: Ablation Analysis for Trajectory Recognition; Table S5: Photoresponse of the bottom-ITO/substrate control under 380, 550 and 660 nm illumination at a 100 mV read bias.

## Abbreviations

The following abbreviations are used in this manuscript:

ITO	Indium tin oxide
RC	Reservoir computing
AFM	Atomic force microscopy
MNIST	Modified National Institute of Standards and Technology database
PPF	Paired-pulse facilitation
ReLU	Rectified linear unit
SGD	Stochastic gradient descent
XPS	X-ray photoelectron spectroscopy
SMU	Source-measure unit
