# Supplementary material for: Multiwavelength Optical Pulse-Induced Temporal State Mapping in an ITO/SnO2/ITO Optoelectronic Synaptic Device
Source: Nanomaterials (Basel). 2026 Oct 8;16(19):1268. doi: 10.3390/nano16191268 (PMC13667160; doi:10.3390/nano16191268)
Supplement: Supplementary file 1 [file nanomaterials-16-01268-s001.zip › nanomaterials-4600657-supplementary.pdf]

Supporting Information

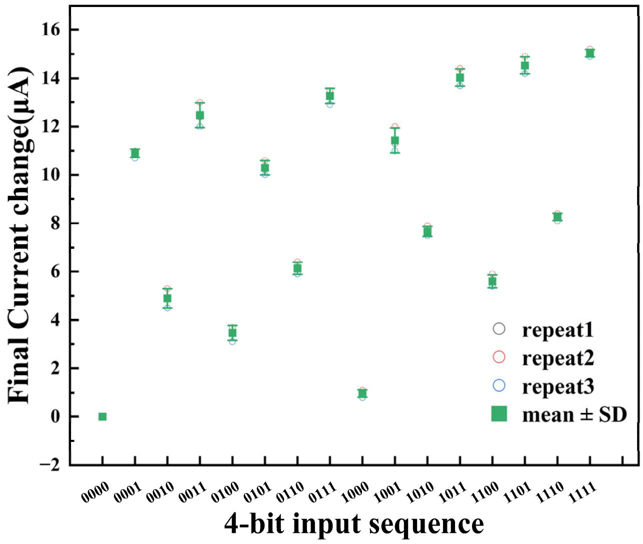

Figure S1a. Within-device repeatability of the 16 four-bit states over three repeated measurements. Individual symbols represent repeated measurements, and error bars denote mean ± SD.

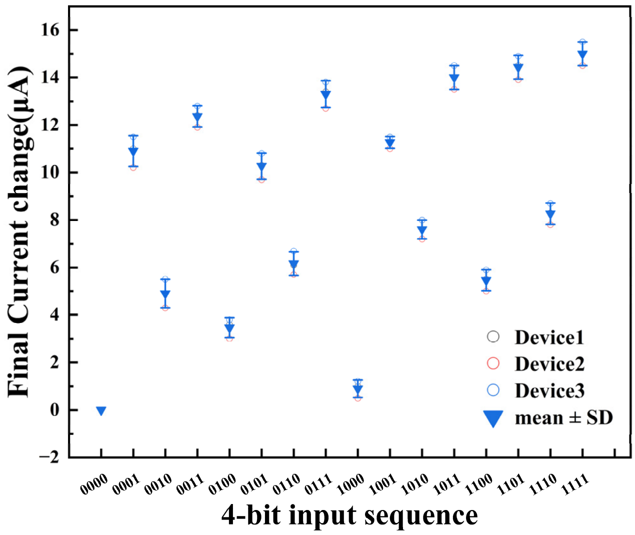

Figure S1b. Device-to-device reproducibility of the 16 four-bit states across three independently fabricated devices. Device 1 is represented by the mean of its three repeated measurements; error bars denote mean ± SD across three devices.

Neighbouring-state separability was assessed by ordering the 16 states according to their mean responses and comparing their mean ± SD intervals. Eleven of the 15 neighbouring pairs showed non-overlapping intervals, while four closely spaced pairs ([0001]/[1001], [0011]/[0111], [1011]/[1101], and [1101]/[1111]) showed partial overlap.

Table S1. Randomized sequence-application orders used in three repeated 16-state measurement cycles.

Measurement order	Cycle 1	Cycle 2	Cycle 3
1	1111	0110	0100
2	1011	1110	0001
3	0101	1011	0101
4	1110	0010	1100
5	0100	0100	0010
6	0000	0001	1111
7	1101	1000	1000
8	0111	1111	0000
9	0011	0111	1001
10	1001	1101	0111
11	0110	1010	0110
12	0001	1100	1011
13	1000	1001	1101
14	1100	0011	1110
15	0010	0101	0011
16	1010	0000	1010

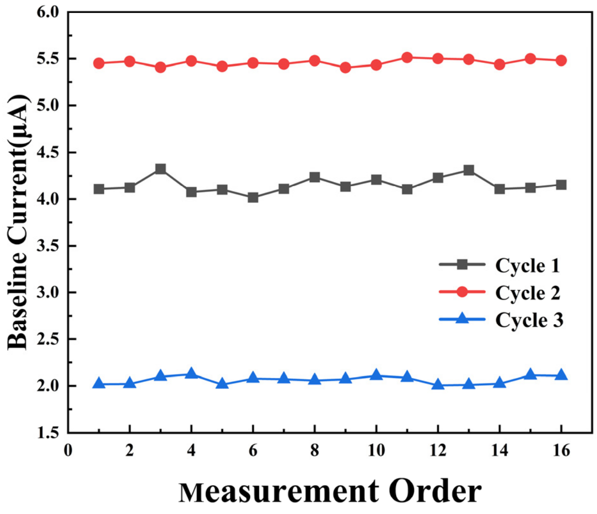

Figure S2. Baseline-current evolution during three randomized 16-state measurement cycles.

Table S2. Wavelength-dependent responsivity and photon-flux-normalized response.

Wavelength (nm)	Power density (mW cm⁻²)	ΔI (μA)	Responsivity (A W⁻¹)	Photon flux (10^{17} photons cm⁻² s⁻¹)	Photon-flux-normalized response [μA/(10^{17} photons cm⁻² s⁻¹)]
380	78.3	5.7	0.927	1.50	3.81
550	78.3	4.7	0.764	2.17	2.17
660	78.3	1.7	0.276	2.60	0.65

The photon flux density was calculated as $\Phi = P\lambda/hc$. For responsivity calculation, the circular top-electrode area (diameter = 0.1 mm) was taken as the active optical area, giving A = 7.85×10^{-5} cm^2. At an incident power density of 78.3 mW cm^{-2}, the corresponding incident optical power was approximately 6.15 μW. The responsivity was calculated as R =ΔI/Pinc. Photon-flux normalization retained the wavelength-dependent response order of 380 > 550 > 660 nm.Repeated dark-current measurements at a 100 mV read bias yielded 12.37 ± 0.26 μA (mean ± SD, n=10), corresponding to a coefficient of variation of 2.09% and a range of 12.0–12.8 μA. These values represent the experimental current fluctuation under the measurement conditions rather than a spectral noise-density measurement.

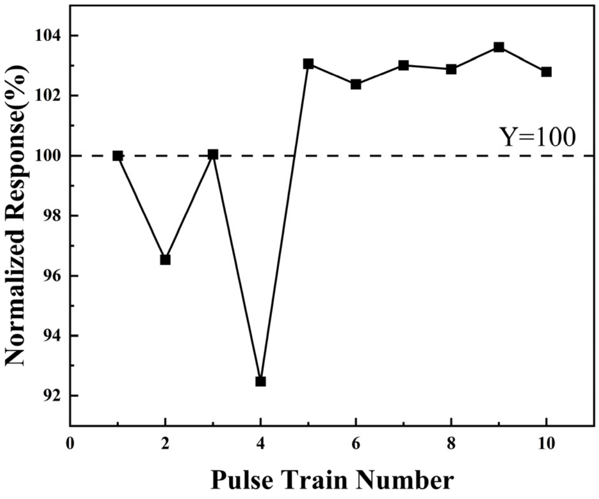

Figure S3a. Normalized final response over 10 repeated optical pulse trains (100 optical pulses in total). Each train consisted of 10 optical pulses followed by recovery to baseline before the next train. The normalized response remained within 92.47–103.61%, with a coefficient of variation of 3.59%; the tenth train retained 102.79% of the first-train response.

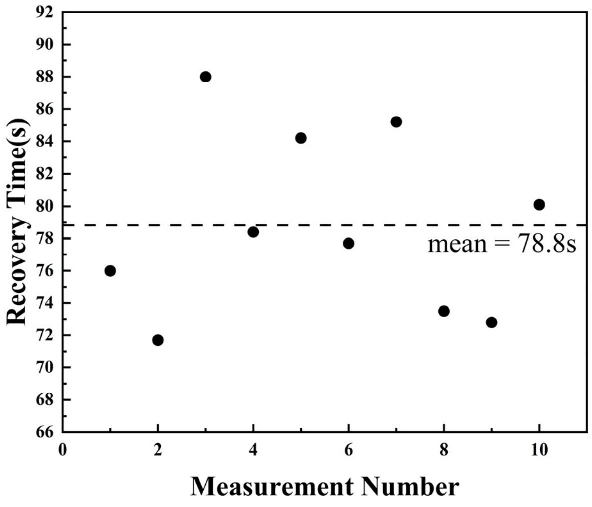

Figure S3b. Recovery times obtained from 10 repeated [1111] stimulation measurements. The average recovery time was 78.8 ± 5.6s (mean ± SD).

Table S3. MNIST baseline comparison. To assess the contribution of the measured device-state mapping, additional MNIST baselines were evaluated using the same dataset split and MLP training protocol. The comparisons included raw binary input, 2 × 2 average pooling, conventional 2 × 2 encoding and non-device-derived software lookup representations. Five independent runs were performed for each representation.

Representation	Input dimension	Test accuracy (%)
Raw binary	784	96.472 ± 0.120
2×2 average pooling	196	96.358 ± 0.116
Conventional 2×2 encoding	196	96.036 ± 0.175
Linear software LUT	196	96.032 ± 0.179
Quadratic software LUT	196	95.420 ± 0.144
Measured device LUT	196	96.062 ± 0.188

Table S4. Ablation Analysis for Trajectory Recognition. Ablation experiments were performed using the same dataset and training protocol as the trajectory-recognition task. Device-state, auxiliary, and combined representations were evaluated separately, together with conventional software temporal and order-insensitive pulse-count encodings. Each condition was evaluated over five independent runs.

Representation	Dimension	Test Accuracy (%)
Device-state features	784	85.10 ± 1.38
Auxiliary features	84	99.90 ± 0.00
Device-state + auxiliary features	868	98.26 ± 0.28
Software 4-bit temporal encoding	784	85.42 ± 0.54
Pulse-count encoding	784	58.20 ± 0.40
Spatial/statistical features	64	62.28 ± 0.96
Centroid-motion features	20	100.00 ± 0.00
Device-state + spatial/statistical features	848	86.44 ± 0.97

Table S5. Photoresponse of the bottom-ITO/substrate control under 380, 550 and 660 nm illumination at a 100 mV read bias.

Wavelength (nm)	ΔI values (μA)	Mean ΔI ± SD (μA)	Relative current change (%)
380	−6, +1, +3	−0.67 ± 4.73	−0.10 ± 0.69
550	+5, −9, +20	5.33 ± 14.50	0.81 ± 2.11
660	−5, +21, +10	8.67 ± 13.05	1.27 ± 1.93
